# 2B-Alert Web 2.0, an Open-Access Tool for Predicting Alertness and Optimizing the Benefits of Caffeine: Utility Study

**DOI:** 10.2196/29595

**Published:** 2022-01-27

**Authors:** Jaques Reifman, Kamal Kumar, Luke Hartman, Andrew Frock, Tracy J Doty, Thomas J Balkin, Sridhar Ramakrishnan, Francisco G Vital-Lopez

**Affiliations:** 1 Department of Defense Biotechnology High Performance Computing Software Applications Institute Telemedicine and Advanced Technology Research Center U.S. Army Medical Research and Development Command Fort Detrick, MD United States; 2 The Henry M. Jackson Foundation for the Advancement of Military Medicine, Inc Bethesda, MD United States; 3 Behavioral Biology Branch Walter Reed Army Institute of Research Silver Spring, MD United States; 4 Oak Ridge Institute for Science and Education Research Participation Program Oak Ridge, TN United States

**Keywords:** alertness-prediction model, caffeine intervention, neurobehavioral performance, psychomotor vigilance test, PVT, sleep loss

## Abstract

**Background:**

One-third of the US population experiences sleep loss, with the potential to impair physical and cognitive performance, reduce productivity, and imperil safety during work and daily activities. Computer-based fatigue-management systems with the ability to predict the effects of sleep schedules on alertness and identify safe and effective caffeine interventions that maximize its stimulating benefits could help mitigate cognitive impairment due to limited sleep. To provide these capabilities to broad communities, we previously released *2B-Alert* Web, a publicly available tool for predicting the average alertness level of a group of individuals as a function of time of day, sleep history, and caffeine consumption.

**Objective:**

In this study, we aim to enhance the capability of the *2B-Alert* Web tool by providing the means for it to *automatically* recommend safe and effective caffeine interventions (time and dose) that lead to optimal alertness levels at user-specified times under any sleep-loss condition.

**Methods:**

We incorporated a recently developed caffeine-optimization algorithm into the predictive models of the original *2B-Alert* Web tool, allowing the system to search for and identify viable caffeine interventions that result in user-specified alertness levels at desired times of the day. To assess the potential benefits of this new capability, we simulated four sleep-deprivation conditions (sustained operations, restricted sleep with morning or evening shift, and night shift with daytime sleep) and compared the alertness levels resulting from the algorithm’s recommendations with those based on the US Army caffeine-countermeasure guidelines. In addition, we enhanced the usability of the tool by adopting a drag-and-drop graphical interface for the creation of sleep and caffeine schedules.

**Results:**

For the 4 simulated conditions, the *2B-Alert* Web–proposed interventions increased mean alertness by 36% to 94% and decreased peak alertness impairment by 31% to 71% while using equivalent or smaller doses of caffeine as the corresponding US Army guidelines.

**Conclusions:**

The enhanced capability of this evidence-based, publicly available tool increases the efficiency by which diverse communities of users can identify safe and effective caffeine interventions to mitigate the effects of sleep loss in the design of research studies and work and rest schedules.

## Introduction

### Background

Previously, we developed and publicly released the *2B-Alert* Web application [1], allowing users to compare and contrast predictions of alertness levels based on the psychomotor vigilance test (PVT) for a group of individuals as a function of time of day, sleep and wake schedule, and caffeine dose [2]. Over the last 15 years, our group at the US Army incrementally developed and enhanced mathematical models that form the core of the *2B-Alert* Web tool. At each developmental step, we created models with additional capabilities and independently validated the model predictions using an array of studies that investigated different sleep-deprivation conditions [3-8]. In total, we have assessed the model predictions for nearly 1200 participants from more than 50 laboratory and field studies. The study conditions ranged from sleep extension (10 h [hours] in bed) to chronic sleep restriction (3-7 h of sleep per night) to total sleep deprivation (TSD; up to 88 h of continuous wakefulness), with several studies performed to investigate the recuperative effects of caffeine administered in both single and multiple doses (50-600 mg), under a variety of sleep and wake schedules.

To date, the *2B-Alert* Web tool has nearly 25,000 registered users from 144 countries, with more than 1800 users from 54 countries accessing the site ≥2 times in the last 12 months and a daily average of 26 log-ins. This is the only publicly available tool of its kind. Here, we describe an enhanced version of the tool, which has the added capability of automatically suggesting caffeine interventions (time and dose) to optimize alertness levels for desired (user-specified) times of day. For any particular combination of user-defined sleep and wake schedule, desired peak-alertness periods, maximum alertness-impairment threshold during these periods, and maximum total caffeine consumption in a 24-h period, the updated tool generates an optimal and safe caffeine-dosing schedule. Specifically, the algorithm generates schedules that use the least amount of caffeine to achieve a user-defined alertness level or achieve *maximum* alertness levels for a user-defined amount of caffeine [9]. This capability would be particularly important during sleep-deprivation conditions because it maximizes the utility of caffeine as a fatigue countermeasure.

### Caffeine-Optimization Algorithm

To attain this functionality, we incorporated a recently developed caffeine-optimization algorithm [9] with the predictive models [7,8] of the original *2B-Alert* Web application [2]. Previously, the tool predicted alertness levels for user-defined sleep and wake and caffeine schedules but required multiple trial-and-error simulations when the user wanted to determine a caffeine schedule that resulted in peak alertness levels during desired wake periods. In the new, enhanced version, this process is performed automatically, leading to a more effective means to identify safe caffeine interventions to guide the design of work and rest schedules and caffeine studies. Using this new capability of the tool, we demonstrated that, compared with the US Army guidelines for the use of caffeine as a countermeasure to sleep deprivation [10], the *2B-Alert* Web–proposed interventions increased mean alertness by 36% to 94% and decreased peak impairment by 31% to 71% while using equivalent or smaller doses of caffeine. In addition, we enhanced the usability of the tool by adopting a drag-and-drop graphical interface for the creation of sleep and caffeine schedules.

## Methods

### Original and Updated Capabilities

The enhanced *2B-Alert* Web tool predicts PVT performance, a measure of alertness and sustained attention, for a typical individual (as determined from group data) as a function of time of day, sleep and wake schedule, and caffeine consumption (dose and time). It offers the capability to accomplish the following:

- Compare and contrast the effects of different sleep and wake and caffeine schedules on alertness.
- Automatically identify caffeine interventions that lead to the desired alertness levels at the desired times of day.

**Figure 1:**
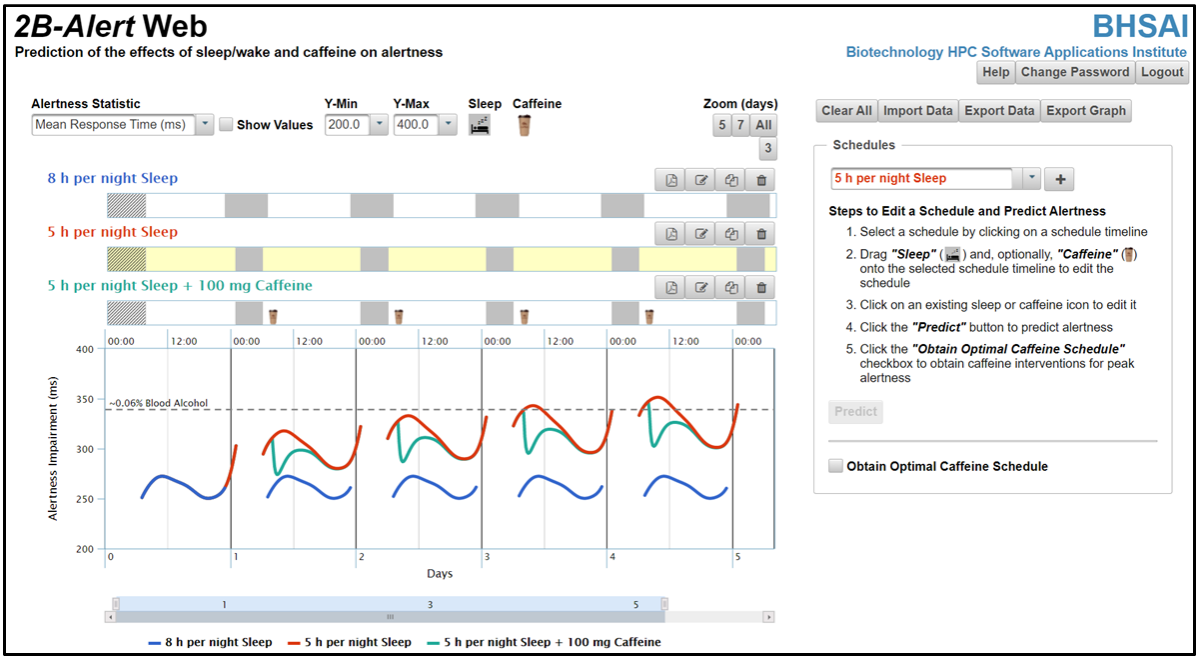
Log-in screen of the *2B-Alert* Web application. This initial screen is preloaded with 3 sleep and caffeine schedules. The yellow background indicates the selected schedule (5 h per night sleep). Users select a schedule by clicking on it and add sleep and caffeine episodes by dragging and dropping the corresponding icon at the top of the screen onto the schedule timeline. The colors of the alertness-impairment prediction plots match those of the names of the corresponding schedules, and users select through a drop-down menu one of three predicted alertness outcome statistics for the psychomotor vigilance test: mean response time (shown), mean speed, or number of lapses >500 ms for a 10-minute psychomotor vigilance test. Users can obtain brief descriptions of the graphical interface functionalities by hovering over the various buttons with the computer mouse. A more comprehensive description is available in the user guide accessible by pressing the Help button at the upper right-hand corner of the page. h: hours.

**Figure 2:**
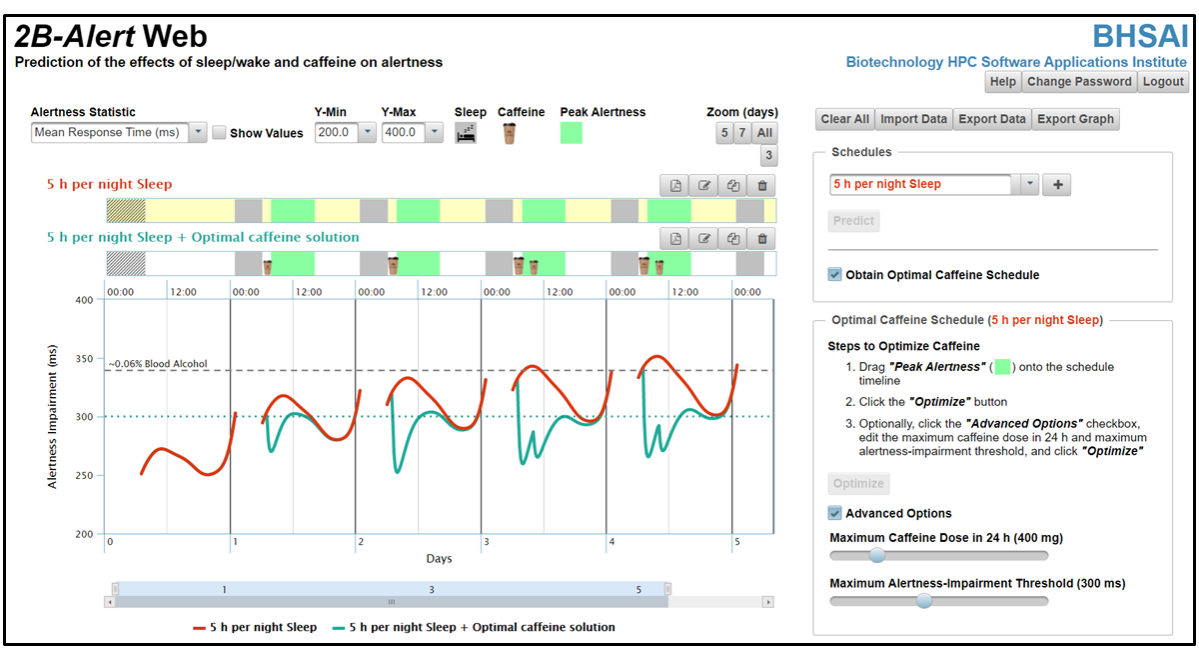
Caffeine-optimization screen. Clicking on the *Obtain Optimal Caffeine Schedule* checkbox at the bottom of the right-hand panel in Figure 1 takes users to the caffeine-optimization screen shown here, and unchecking the box returns users to the prediction screen in Figure 1. From this screen, users can define periods of peak alertness by dragging and dropping the green icon at the top of the page onto the selected schedule and obtain caffeine schedules that result in desired alertness levels for the user-specified peak-alertness periods. The Advanced Options checkbox (lower right-hand panel) allows users to specify thresholds in the optimization algorithm for total caffeine in a running 24-h period and alertness-impairment levels during periods of peak alertness. h: hours.

For the former capability, which is also offered in the original tool [2], it provides alertness-level (ie, PVT) predictions for a given sleep and wake and caffeine schedule (Figure 1). Now, the updated tool offers the optional capability to automatically identify the optimal time and dose of caffeine consumption that lead to the desired, user-defined alertness outcomes. For (1) a specified sleep and wake schedule, (2) desired periods of peak alertness, (3) maximum acceptable alertness-impairment threshold during peak-alertness periods, and (4) maximum total caffeine consumption over a 24-h running period, the tool provides caffeine timing and dosing suggestions to achieve peak alertness at the desired times to the extent allowed by the limit on caffeine consumption (Figure 2).

### Overview of the 2B-Alert Web Tool

Upon user log-in, the tool provides brief stepwise instructions on how to select, edit, and add sleep and caffeine episodes to a schedule and make predictions (Figure 1, right-hand panel). Similarly, when the user navigates to the caffeine optimization screen by clicking the *Obtain Optimal Caffeine Schedule* checkbox at the bottom of the right-hand panel in Figure 1, the tool also provides brief stepwise instructions on how to create peak alertness periods, obtain an optimal caffeine schedule, and how to edit the alertness-impairment threshold and the maximum total caffeine consumption over a 24-h running period (Figure 2, right-hand panel). In addition, users can obtain brief descriptions of the functionalities of the various interaction-enabled buttons of the graphical interface by hovering over the buttons with a mouse. When the user clicks on the *Help* button in the upper right-hand corner of the display window, the system displays a user guide, which provides a comprehensive description of the functionalities of the system to facilitate assimilation of the *2B-Alert* Web tool.

### Comparing and Contrasting Different Sleep and Caffeine Schedules

The *2B-Alert* Web tool can be used to compare and contrast the effects of different schedules on alertness. A schedule is defined by a series of sleep episodes and caffeine episodes spanning a period of up to 30 days (note that all day and time entries are for the same time zone). In turn, a sleep episode is defined by start and end days and times, whereas a caffeine episode is defined by the day, time, and dose of caffeine, with the dose entered manually or selected from a drop-down list of more than 30 popular caffeine-containing products. Upon user log-in, the tool displays three preloaded schedules: *8 h per night sleep*, *5 h per night sleep*, and *5 h per night sleep + 100 mg caffeine*, with the middle schedule (yellow background) selected, as shown in the screenshot of the *2B-Alert* Web tool interface (Figure 1).

The user can modify an existing schedule or create a new schedule. For an existing schedule, users may view and edit the day and time of sleep episodes (gray icons) as well as caffeine-dose episodes (cup icons) by clicking on the corresponding icon. For example, when the user clicks on the sleep icon of the selected schedule in Figure 1 (*5 h per night sleep*), a pop-up window displays the start and end days of the sleep episode, along with the start (01:00) and end (06:00) times. Users may also add sleep and caffeine episodes by dragging and dropping the corresponding icon located above the schedules onto the timeline of the selected schedule. Users may select a schedule by clicking on its timeline or by choosing it from the drop-down list of preloaded timelines under *Schedules* on the right-hand side of the figure. The four buttons, from left to right, on the upper right-hand side of each schedule timeline allow users to export the schedule as a PDF file, change the schedule name, save a schedule with the same or a different name, or delete the schedule, respectively.

To create a new schedule, the user clicks the plus button to the right of the Schedule drop-down menu (Figure 1, right-hand panel) and then adds sleep and caffeine episodes. Alternatively, a schedule can be imported from a Microsoft Excel file (Figure 1, *Import Data*) using a predefined format. Each generated or edited schedule can be saved with a user-defined name and exported as a PDF file (Figure 1, left-most of the 4 buttons on the upper right-hand side of this schedule’s timeline). The system supports up to 5 schedules and plots per session, where users can hide (or unhide) plots from view by clicking on the corresponding schedule name below the x-axis scroll bar. If the interface is loaded with 5 schedules, to create a new schedule, the user should delete an existing one.

The predicted alertness impairment for each schedule can be compared in the graph below the schedules. Figure 1 shows the 3 corresponding predictions for the 3 preloaded schedules using the mean response time (RT; in ms) PVT statistic, from *day 0* through *day 4* of the 4-day schedule, which starts on *day 1* and lasts until the start of the sleep episode on *day 5*. The plots allow for the comparison of the effects of different sleep durations on alertness (eg, 5 h vs 8 h of sleep per night), as well as for the assessment of the beneficial effects of caffeine countermeasures (eg, 5 h per night of sleep with and without 100 mg of daily caffeine at 08:00). The displayed plots can be saved in an image file using the *Export Graph* button, and the numerical values for each of the 3 predicted statistics can be exported into an Excel file, along with the corresponding sleep and wake and caffeine schedules (Figure 1, *Export Data*). This allows users to import and reuse schedules in a future session because the system does not save schedules or their predictions on the web (the system erases all data when the user logs out).

In addition to the mean RT, the user can select to display the plots of alertness-impairment predictions for two other PVT statistics: mean speed (average of the reciprocal of RT; in 1/s) and number of lapses (number of RTs >500 ms). The statistics are for a 10-minute test and are selected from the *Alertness Statistic* drop-down menu located above the schedules (Figure 1). To map PVT statistics into a more broadly understood metric of vigilance deficits, we used the findings from Dawson and Reid [11] and Williamson et al [12] to obtain an equivalence between PVT alertness-impairment values and blood alcohol concentrations (BACs). We estimated that a mean RT of 339 ms attained after 19 h of continued wakefulness corresponded to a 0.06% BAC (Figure 1, horizontal dashed gray line) and that a mean RT of 458 ms attained after 24 h of continuous wakefulness corresponded to a 0.08% BAC. For context, driving at BACs of 0.06% and 0.08% increase the risk of causing a traffic accident by 2- and 3-fold, respectively, compared with control drivers [13,14].

### Automatic Identification of Caffeine Interventions

To automatically identify caffeine interventions that lead to desired alertness levels for a selected sleep and wake schedule, the user should click the *Obtain Optimal Caffeine Schedule* checkbox at the bottom of the right-hand panel in Figure 1, which takes users to another graphical interface (Figure 2). In this interface, the user can add a desired period of peak alertness (start and end days and times), which is a required input, to the schedule. This is achieved by dragging and dropping the *Peak Alertness* icon (green icon) located above the schedules onto the timeline (Figure 2). Next, clicking the *Optimize* button in the lower right-hand panel will generate the optimal caffeine schedule and display the corresponding alertness-impairment prediction. For the selected schedule shown in Figure 1 (*5 h per night sleep*), these steps resulted in the identification of the optimal caffeine intervention named *5 h per night Sleep + Optimal caffeine solution* in Figure 2 and the corresponding alertness-impairment prediction plot. In this case, peak-alertness periods from 08:00 to 16:00 for *day*s *1* to *4* required a single caffeine dose at 07:00 of 100 mg on *day 1* and 200 mg on *day 2* and two doses at 07:00 and 10:00 of 200 mg and 100 mg, respectively, on *day*s *3* and *4* (see Figure S1 in Multimedia Appendix 1 for details; larger cup icons indicate larger caffeine doses). Optionally, the *Advanced Options* checkbox at the bottom of the lower right-hand panel allows users to set thresholds for maximum total caffeine in a 24-h period (100-1500 mg; default 400 mg) and the maximum alertness-impairment level (RT ranging from 150-500 ms; default 300 ms) by dragging the sliders to the desired values (Figure 2). As described previously, users can rename, save, delete, and export the updated schedule, as well as save the displayed plot as an image file. Unchecking the *Obtain Optimal Caffeine Schedule* in the upper right-hand panel takes users back to the prediction graphical interface (Figure 1).

### Initial Conditions and Assumptions

We formulated the predictive model in the *2B-Alert* Web tool so that alertness is an inversely related function of sleep debt, which accumulates over days with <8 h of sleep per day [5,7,8] but decreases for sleep durations of >8 h per day. We initialized the tool so that on day *0* there is no sleep debt after 8 h of sleep (23:00-07:00). However, if sleep debt is nonzero, users need to enter up to 7 days of sleep history at the beginning of the schedule (sleep episodes >7 days old have negligible influence on near-future alertness [5,7,8]).

We assumed that the restorative effect of caffeine depends on the alertness level; that is, for a given caffeine dose, the larger the alertness impairment, the greater the beneficial effect of caffeine [15,16], where the magnitude of the benefit depends on the impairment level, the caffeine dose, and the residual concentration of caffeine from previous doses.

For the automatic identification of caffeine interventions that optimize alertness levels, we formulated the optimization problem so that, for the desired periods of peak alertness, the algorithm seeks caffeine-intervention solutions that equally weigh the cumulative deficit above the maximum alertness-impairment threshold and the peak alertness-impairment level [9]. To obtain practical and safe solutions, we added the following constraints: (1) caffeine doses to be restricted to 100 mg, 200 mg, or 300 mg; (2) dosing to occur on the hour; (3) the minimum time period between doses to be 2 h; and (4) the cumulative caffeine concentration in the blood to be below the maximum level achieved by a single 400-mg dose [17]. The optimization algorithm finds solutions in a matter of seconds by generating and assessing only caffeine schedules that are likely to reduce alertness impairment [9]. The tool attempts to find solutions that use the least amount of caffeine, while meeting the imposed constraints. However, in certain cases, even when using the maximum amount of caffeine, it may not be possible to obtain solutions that reduce the alertness impairment below the maximum impairment threshold. To obtain solutions with lower alertness impairment, users may increase the maximum total caffeine consumption in a 24-h period (Figure 2, *Advanced Options*). Alternatively, the near-optimal *2B-Alert* Web solution provides a good starting point for manual exploration of additional caffeine interventions that are potentially more effective but likely violate specified constraints.

### Simulations to Assess the Optimization Algorithm

**Figure 3:**
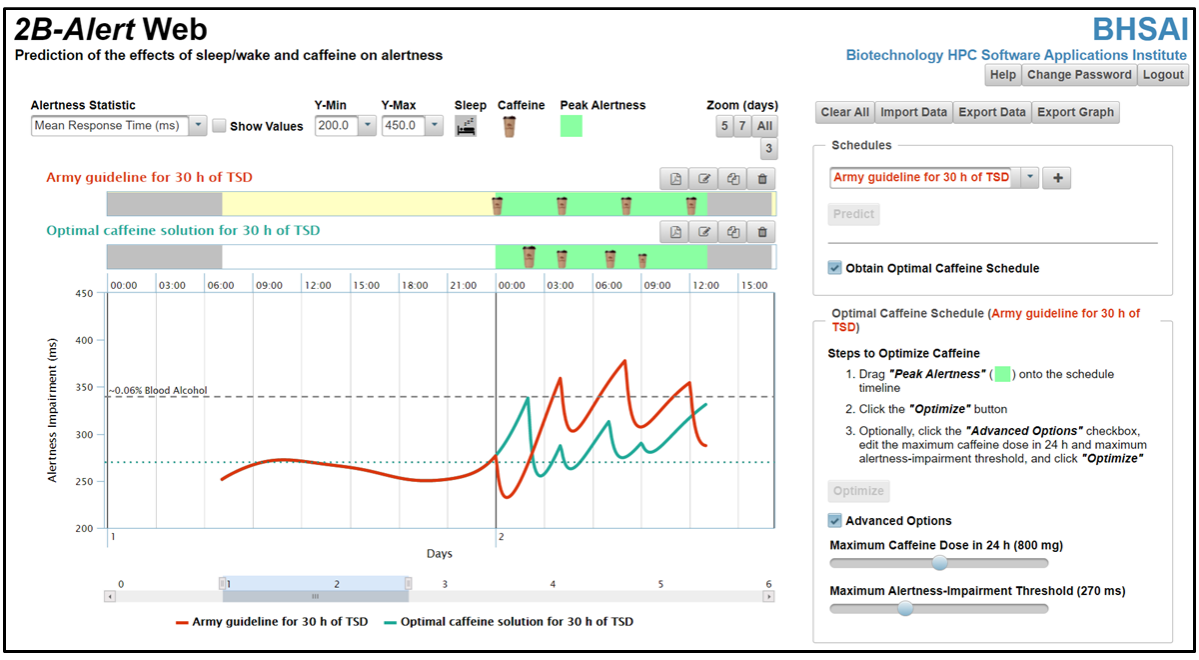
*2B-Alert* Web tool versus US Army caffeine recommendations for sustained operations (condition 1 in Table 1). Comparison of the effects of caffeine countermeasures as recommended by the US Army guidelines (top schedule) versus those automatically identified by the *2B-Alert* Web tool (bottom schedule) for 30 h of TSD with a user-defined peak alertness period ranging from midnight to 13:00 on day 2. Although neither solution was capable of maintaining alertness below the selected 270-ms impairment threshold with 800 mg of caffeine, the tool’s solution (green line) avoided impairment levels surpassing the 0.06% blood alcohol concentration equivalence level. During the 13 h of peak alertness, it also reduced the mean alertness impairment by 56% and the peak alertness impairment by 36% (Table 2). h: hours; TSD: total sleep deprivation.

To assess the efficacy and potential benefits of the automated caffeine interventions proposed by the *2B-Alert* Web algorithm, we performed 4 simulations encompassing the three circumstances under which caffeine countermeasures are considered by the US Army guidelines [10]: sustained operations, restricted sleep, and night shift. Table 1 summarizes the US Army guidelines for the use of caffeine as a countermeasure to sleep deprivation and the simulated conditions, including the sleep-deprivation schedules and the assumed periods of peak alertness. For example, for the sustained operations scenario in condition 1, we simulated a 30-h TSD challenge from 07:00 on *day* *1* to 13:00 on *day* *2* and arbitrarily assumed a 13-h peak-alertness period from 00:00 to 13:00 on *day* *2* and a maximum alertness-impairment threshold of 270 ms (Figure 3), which corresponds to the maximum alertness impairment under well-rested conditions (ie, habitual sleep of 8 h per day). Then, we used the tool to obtain an optimal caffeine schedule and compared the resulting alertness impairment with that obtained by using the US Army guidelines. Figures S2-S7 in Multimedia Appendix 1 provide details for the simulations of conditions 2-4.

**Table 1:** Summary of the US Army guidelines for the use of caffeine as a countermeasure to sleep deprivation, simulated conditions (including type of sleep challenge, sleep schedule, and desired period of peak alertness), and recommended caffeine countermeasures based on the guidelines and the *2B-Alert* Web optimization algorithm.

Condition		Army caffeine guideline	Sleep schedule	Peak-alertness period	Countermeasure recommendation	
					Army	*2B-Alert* Web
1. Sustained operations: 30 h^a^ of total sleep deprivation		200 mg every 4 h as needed, starting at 00:00	N/A^b^	00:00-13:00 on day 2	800 mg in 4 doses (Figure 4A)	800 mg in 4 doses (Figure 4B)
2. Chronic sleep restriction: morning shift		200 mg upon awakening, 200 mg 4 h later	01:00-06:00 for 5 days	08:00-16:00 for 5 days	2000 mg in 10 doses (Figure S3A^c^)	1900 mg in 13 doses (Figure S3B^c^)
3. Chronic sleep restriction: evening shift		200 mg upon awakening, 200 mg 4 h later	01:00-06:00 for 5 days	15:00-23:00 for 5 days	2000 mg in 10 doses (Figure S5A^c^)	1700 mg in 11 doses (Figure S5B^c^)
4. Night shift with daytime sleep		200 mg at the beginning of the shift	20:00 to 22:00 and 10:00 to 15:00 for 5 days	00:00 to 08:00 for 5 days	1000 mg in 5 doses (Figure S7A^c^)	1000 mg in 5 doses (Figure S7B^c^)

^a^h: hours.

^b^N/A: not applicable.

^c^See Figures S3, S5, and S7 in Multimedia Appendix 1.

### Alertness Improvement Metrics

To quantify the potential benefit of the caffeine-optimization algorithm, we used two metrics: mean alertness impairment and peak alertness impairment, each computed over the desired periods of peak alertness. The mean alertness impairment accounts for the overall impairment level across the peak alertness periods, whereas the peak alertness period captures acute levels of impairment over short periods of time during the peak alertness periods. For the mean RT PVT metric used in our simulations, we defined the mean alertness impairment (in ms) as the ratio of the area of the predicted alertness impairment above the selected maximum alertness-impairment threshold to the duration of the peak alertness periods. Similarly, we defined the peak alertness impairment (in ms) as the maximum mean RT over all periods of desired peak alertness minus the selected maximum alertness-impairment threshold. In addition, we computed the total time (in h) that the predicted alertness impairment was above the 0.06% BAC threshold during the peak alertness periods as a measure of time for which an individual is at an increased risk of making a mistake because of deficits in alertness.

### System Architecture

The overall software architecture of the updated *2B-Alert* Web tool was unchanged from the original release except for the overhaul of the graphical user interface and the use of updated versions of the underlying software technologies. We hosted the tool on an Apache Tomcat web server and provided access through a secure service over HTTP Secure. We used a three-tier architecture consisting of (1) a PostgreSQL database server, which stores user account information; (2) a controller, which provides access to the alertness-prediction model and caffeine-optimization algorithm and implements the functionalities required to create and manage multiple predictions and optimizations; and (3) an interaction-enabled user interface, which provides the ability to create schedules by dragging and dropping sleep, caffeine, and peak-alertness episodes onto the schedule timeline; show and hide plots; and dynamically zoom into and out of plots. The system runs without any plugins and is accessible through multiple web browsers, including Internet Explorer version 11, Chrome version 74, and Firefox version 67, or earlier versions thereof.

### Access and Privacy

The *2B-Alert* Web tool is freely available to registered users through a secure web browser. Registration consists of users providing their name, email address, and affiliation, after which they receive a confirmation email with a username and password, which can be changed. The system erases all simulated schedules and results when the user logs out, providing user privacy and maintaining data confidentiality.

## Results

### Potential Benefits of the 2B-Alert Web Caffeine-Optimization Algorithm

**Figure 4:**
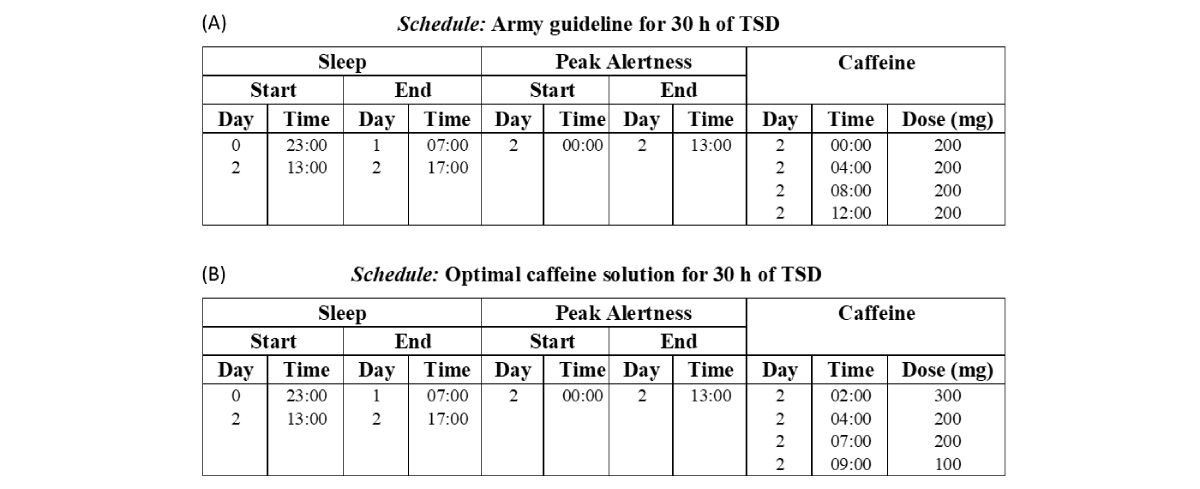
Sleep schedule, peak-alertness schedule, and caffeine recommendations for the results depicted in Figure 3: (A) US Army guidelines and (B) optimal caffeine solution automatically generated by the *2B-Alert* Web tool. Users export this information as PDF files by clicking on the left-most of the 4 buttons on the upper right-hand side of each schedule’s timeline in Figure 3. h: hours; TSD: total sleep deprivation.

To demonstrate the potential benefit of the *2B-Alert* Web caffeine-optimization algorithm, we compared the effects of caffeine countermeasures as recommended by the US Army guidelines [10] with those of the tool for the 4 conditions illustrated in Table 1. For the sustained-operations scenario in condition 1 (Figure 3), the guideline recommended an initial 200-mg caffeine dose at midnight, followed by 200 mg every 4 h thereafter (at 04:00, 08:00, and 12:00) for a total of 800 mg over a 12-h span (Figure 4A). Figure 3 shows the resulting alertness-impairment prediction plot in terms of the mean RT (red line). Figure 3 also shows the optimal caffeine schedule recommended by the tool and the associated alertness-impairment prediction plot (green line) for the same total amount of 800 mg of caffeine. Figure 4B, obtained by exporting the *Optimal caffeine solution for 30 h of TSD* schedule as a PDF, shows the numerical values of the optimal caffeine times and doses recommended by the tool. By suggesting caffeine interventions at different time intervals in varying doses of 100 mg, 200 mg, or 300 mg over a more compressed 7-h span during which time the circadian-mediated alertness deficits were greatest, the *2B-Alert* Web solution consistently kept impairment below the 0.06% BAC equivalence level during the entire peak-alertness period. In contrast, the US Army guideline solution was less effective, resulting in 3 time periods when alertness deficits exceeded the 0.06% BAC level for a total of 3.4 h above this threshold and impairment levels as high as 378 ms (270+108 ms) around 08:00 on *day 2* (Table 2 and Figure 3). To provide a context of the significance of this difference, an individual following the US Army guidelines would have a 2-fold increased risk of causing a traffic accident during the 3.4-h period compared with substantially lower increase in risk with the tool’s recommendations. Nevertheless, for a maximum of 800 mg of caffeine, neither recommendation was capable of maintaining alertness levels below the maximum user-defined threshold of 270 ms for most of the 13-h period of peak alertness.

**Table 2:** Predicted alertness impairment during the selected peak-alertness periods for the 4 simulated sleep-challenge conditions in using caffeine recommendations from the US Army guidelines and the *2B-Alert* Web optimization algorithm. Both the mean alertness impairment and the peak alertness impairment are computed considering impairment above the user-specified maximum alertness-impairment threshold. Although the mean impairment was averaged over the corresponding peak-alertness periods in the simulated days, the peak impairment values correspond to the maximum impairment over the same periods. The tool improved the mean alertness on average by 59% (SD 25%) and peak alertness by 45% (SD 18%). The time durations for which the predictions reached impairment levels above the 0.06% blood alcohol concentration (BAC) equivalent are provided to help with interpretation of the results.

Condition	Mean alertness impairment (ms)		Improvement (%)	Peak alertness impairment (ms)			Improvement (%)		Time above 0.06% BAC equivalent (h)	
	Army	*2B-Alert* Web		Army	*2B-Alert* Web			Army		*2B-Alert* Web
1	48	21	56	108	69	36		3.4		0.0
2	8	4	50	44	25	43		0.0		0.0
3	18	1	94	38	11	71		0.0		0.0
4	45	29	36	126	87	31		14.6		3.1

### Caffeine Recommendations

Similarly, the *2B-Alert* Web caffeine recommendations for the sleep restriction conditions and the night shift with daytime sleep condition in Table 1 yielded superior results when compared with the US Army guidelines solutions. The tool’s recommendations yielded larger improvements in mean alertness impairment and peak alertness impairment, while using the same or as much as 300 mg less caffeine than the US Army’s recommendations (Tables 1 and 2). In condition 2, although the percentage improvement in mean alertness impairment was 50%, the absolute improvement averaged over the peak-alertness periods in the 5 simulated days was minimal (4 ms vs 8 ms). Given the limited amount of sleep deficit, the relatively narrow period of desired peak alertness (8 h per day), and the relatively large amount of consumed caffeine per day (400 mg), there were not enough degrees of freedom in this problem to demonstrate a considerable improvement of the optimization algorithm over the guidelines solution because it already provided near-optimal benefits. For related reasons, at times, the benefits achieved over peak-alertness periods came at the detriment of other wakefulness periods (eg, condition 3). Figures S2-S7 in Multimedia Appendix 1 provide detailed results for the simulations of conditions 2-4. Overall, our simulation results show that customized caffeine recommendations intended to enhance alertness during a specific time of the day consistently resulted in higher levels of alertness compared with suitable but generic recommendations.

## Discussion

### Principal Findings

We have enhanced the publicly available *2B-Alert* Web tool to automatically identify caffeine interventions that safely optimize alertness within selected time periods for any user-specified sleep and wake schedule. Users can use the tool to compare and contrast the effects of different sleep and wake and caffeine schedules on the alertness of a group of individuals and to automatically identify safe caffeine interventions to achieve and sustain desired alertness levels for desired times of day. To achieve this added capability, we integrated a well-validated mathematical model, which predicts alertness impairment levels based on the PVT as a function of time of day, sleep and wake schedule, and caffeine consumption, with a recently developed caffeine-optimization algorithm, which identifies the minimum amount of caffeine required to restore alertness levels, while imposing multiple operational and safety constraints (some of which can be modified by the user). The *2B-Alert* Web tool synthesizes decades of sleep-physiology and mathematical modeling research by the US Army into an open-access practical tool that should find value in research and operational communities.

To demonstrate the potential benefits of automatically identifying caffeine recommendations that safely optimize alertness for desired time periods, we simulated four conditions of military relevance reflective of sustained operations, restricted sleep (with morning and evening shifts), and night shift with daytime sleep (Table 1). Next, we compared the resulting alertness impairment levels by considering the US Army guidelines for the use of caffeine as a countermeasure to sleep deprivation [10] against those automatically obtained by using the newly implemented caffeine optimization capability [9] provided by the tool. The results show that the tool consistently provides solutions with greater improvements in mean alertness impairment (by 36% to 94%; mean 59%, SD 25%) and peak alertness impairment (by 31% to 71%; mean 45%, SD 18%), while using the same or as much as 300 mg less caffeine than the US Army’s recommendations (Table 2). Although the extent of such benefits varies as a function of sleep and wake schedule, time and duration of desired peak alertness periods, acceptable alertness-impairment threshold, and maximum total amount of caffeine consumption, the results also demonstrate that the customized caffeine recommendations provided by this new capability are superior to generic, *one-size-fits-all* guidelines.

### Limitations

The tool has limitations. First, the predictive models that form the core of the *2B-Alert* Web application have only been validated for healthy, young adults of military age. The extent to which the tool is applicable to an older, heterogeneous cohort is not known. Second, the tool’s predictions of alertness impairment are in fact surrogates for PVT statistics. Hence, whether such predictions also serve as surrogates for other neurocognitive measures of performance impairment remains to be investigated. Third, all sleep and caffeine times are assumed to be for the same time zone. The ability to predict alertness impairment involving transmeridian travel would require circadian-phase data across time zones, which we do not possess. Fourth, the *2B-Alert* Web tool does not account for the potential disruptive effect of caffeine when consumed soon before sleep episodes. In a future implementation, we could address this limitation by forbidding caffeine recommendations during certain times of the day. Fifth, the predictive models do not account for tolerance to caffeine. However, a sensitivity analysis of the caffeine-model component suggests that changes in model parameters to account for this effect have a very small impact on the predicted alertness [8]. Importantly, experimental observations indicate that the main determinant of the effect of caffeine on alertness is the impairment level, regardless of the caffeine tolerance level [15,16], which is consistent with the model predictions.

Sixth, the tool has not been prospectively tested in military or civilian settings such as the Marine Corps Officer Basic School or shiftwork at hospitals, and we estimated the benefits of the optimal caffeine schedules for the 4 simulation scenarios in Tables 1 and 2 using the tool’s prediction models instead of using actual PVT measurements from an experimental study. However, the mathematical models at the core of the tool have been thoroughly validated in dozens of different sleep and caffeine-consumption conditions. For example, in one of the validation analyses, Ramakrishnan et al [7] showed that approximately 87% of the model predictions are within 2 SEs of the measured mean data, meaning that, 87% of the time, the model group-average predictions are statistically indistinguishable from the experimental data. Thus, we have reason to believe that the model will provide good predictions in conditions for which experimental data are not available, producing adequate guidance for sleep and caffeine schedules when it is not possible or desirable to conduct a study. Finally, we limited predictions to a maximum of 30 consecutive days to be able to provide solutions in a few (less than approximately 3) minutes. Nevertheless, the user can run longer scheduling scenarios by carefully breaking the problem into smaller chunks.

### Conclusions

The new and unique capability to automatically identify caffeine interventions that safely optimize alertness transforms the *2B-Alert* Web tool from an alertness and performance *prediction* tool into an alertness and performance *enhancement* tool. This functionality not only results in peak alertness levels during desired wake periods but, by recommending the right caffeine doses at correct times, also maximizes the utility of caffeine. The *2B-Alert* Web tool provides caffeine schedules that use the least amount of caffeine to achieve a desired alertness level, thus reducing potential side effects of excessive caffeine use, or that yield *maximum* alertness levels for a given amount of caffeine, thus optimizing its recuperative effects.

## Appendix

Multimedia Appendix 1 Supporting information.

## Abbreviations

BAC: blood alcohol concentration
h: hours
PVT: psychomotor vigilance test
RT: response time
TSD: total sleep deprivation
