# Supplementary material for: 2B-Alert Web 2.0, an Open-Access Tool for Predicting Alertness and Optimizing the Benefits of Caffeine: Utility Study
Source: J Med Internet Res. 2022 Jan 27;24(1):e29595. doi: 10.2196/29595 (PMC8832274; doi:10.2196/29595)
Supplement: Multimedia Appendix 1 [file jmir_v24i1e29595_app1.doc]

***2B-Alert* Web 2.0: An Open-Access Tool for Predicting Alertness and Optimizing the Benefits of Caffeine**

Jaques Reifman, PhD^1^; Kamal Kumar, MS^1,2^; Luke Hartman, BS^1,2^; Andrew Frock, BS^1,2^; Tracy J. Doty, PhD^3^; Thomas J. Balkin, PhD^3,4^; Sridhar Ramakrishnan, PhD^1,2^; Francisco G. Vital-Lopez, PhD^1,2^

^1^Department of Defense Biotechnology High Performance Computing Software Applications Institute, Telemedicine and Advanced Technology Research Center, U.S. Army Medical Research and Development Command, Fort Detrick, MD; ^2^The Henry M. Jackson Foundation for the Advancement of Military Medicine, Inc., Bethesda, MD; ^3^Behavioral Biology Branch, Walter Reed Army Institute of Research, Silver Spring, MD; ^4^Oak Ridge Institute for Science and Education, Research Participation Program, Oak Ridge, TN

SUPPORTING INFORMATION

| *Schedule:* 5 h per night Sleep + Optimal caffeine solution   | **Sleep** | | | | **Peak Alertness** | | | | **Caffeine** | | | | --- | --- | --- | --- | --- | --- | --- | --- | --- | --- | --- | | **Start** | | **End** | | **Start** | | **End** | | | **Day** | **Time** | **Day** | **Time** | **Day** | **Time** | **Day** | **Time** | **Day** | **Time** | **Dose (mg)** | | 1 | 01:00 | 1 | 06:00 | 1 | 08:00 | 1 | 16:00 | 1 | 07:00 | 100 | | 2 | 01:00 | 2 | 06:00 | 2 | 08:00 | 2 | 16:00 | 2 | 07:00 | 200 | | 3 | 01:00 | 3 | 06:00 | 3 | 08:00 | 3 | 16:00 | 3 | 07:00 | 200 | | 4 | 01:00 | 4 | 06:00 | 4 | 08:00 | 4 | 16:00 | 3 | 10:00 | 100 | | 5 | 01:00 | 5 | 06:00 |  |  |  |  | 4 | 07:00 | 200 | |  |  |  |  |  |  |  |  | 4 | 10:00 | 100 | |
| --- | --- | --- | --- | --- | --- | --- | --- | --- | --- | --- | --- | --- | --- | --- | --- | --- | --- | --- | --- | --- | --- | --- | --- | --- | --- | --- | --- | --- | --- | --- | --- | --- | --- | --- | --- | --- | --- | --- | --- | --- | --- | --- | --- | --- | --- | --- | --- | --- | --- | --- | --- | --- | --- | --- | --- | --- | --- | --- | --- | --- | --- | --- | --- | --- | --- | --- | --- | --- | --- | --- | --- | --- | --- | --- | --- | --- | --- | --- | --- | --- | --- | --- | --- | --- | --- | --- | --- | --- | --- | --- | --- | --- | --- | --- | --- | --- |

**Figure S1.** Sleep schedule, peak-alertness schedule, and caffeine recommendation for the “5 h per night Sleep + Optimal caffeine solution” in Figure 2. Users export this information as a Portable Document Format file by clicking on the left-most of the four buttons on the upper right-hand side of this schedule’s timeline in Figure 2.


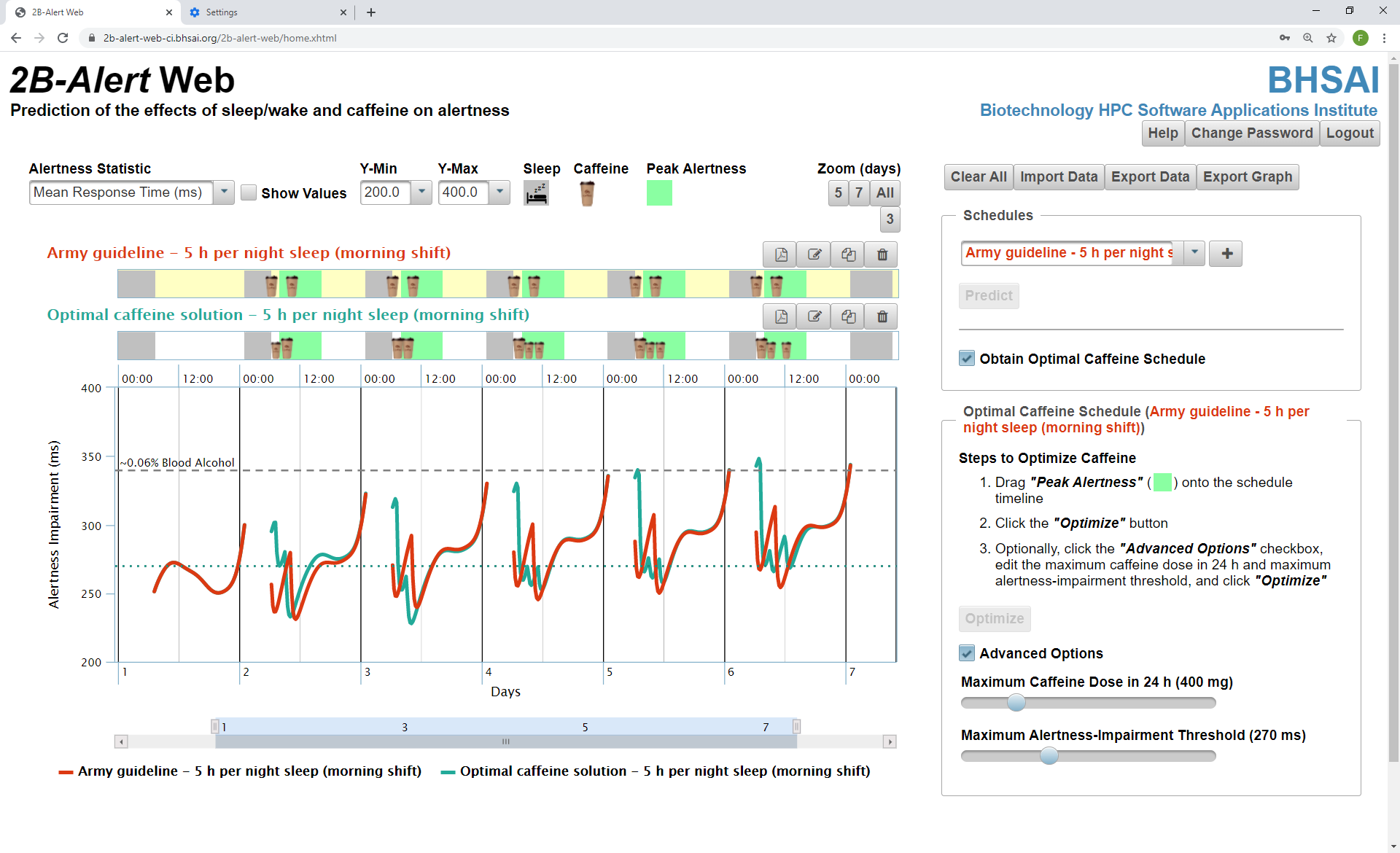


**Figure S2.** *2B-Alert* Web vs. U.S. Army caffeine recommendations for sleep restriction with morning work shift (Condition *2* in Table 1). Comparison of the effects of caffeine countermeasures as recommended by the U.S. Army guidelines (top schedule) vs. those automatically identified by the *2B-Alert* Web tool (bottom schedule) for 5 h per night sleep for 5 nights, with a user-defined peak-alertness period ranging from 08:00 to 16:00 each day. When compared to the U.S. Army guideline, for this peak-alertness period, the optimal caffeine solution provided by the *2B-Alert* Web tool yielded alertness predictions that reduced the mean alertness impairment level by 50% and the peak alertness impairment level by 43% (Table 2).

| (A) Schedule: Army guideline - 5 h per night sleep (morning shift)  | **Sleep** | | | | **Peak Alertness** | | | | **Caffeine** | | | | --- | --- | --- | --- | --- | --- | --- | --- | --- | --- | --- | | **Start** | | **End** | | **Start** | | **End** | | | **Day** | **Time** | **Day** | **Time** | **Day** | **Time** | **Day** | **Time** | **Day** | **Time** | **Dose (mg)** | | 0 | 23:00 | 1 | 07:00 | 2 | 08:00 | 2 | 16:00 | 2 | 06:00 | 200 | | 2 | 01:00 | 2 | 06:00 | 3 | 08:00 | 3 | 16:00 | 2 | 10:00 | 200 | | 3 | 01:00 | 3 | 06:00 | 4 | 08:00 | 4 | 16:00 | 3 | 06:00 | 200 | | 4 | 01:00 | 4 | 06:00 | 5 | 08:00 | 5 | 16:00 | 3 | 10:00 | 200 | | 5 | 01:00 | 5 | 06:00 | 6 | 08:00 | 6 | 16:00 | 4 | 06:00 | 200 | | 6 | 01:00 | 6 | 06:00 |  |  |  |  | 4 | 10:00 | 200 | | 7 | 01:00 | 7 | 09:00 |  |  |  |  | 5 | 06:00 | 200 | |  |  |  |  |  |  |  |  | 5 | 10:00 | 200 | |  |  |  |  |  |  |  |  | 6 | 06:00 | 200 | |  |  |  |  |  |  |  |  | 6 | 10:00 | 200 | |
| --- | --- | --- | --- | --- | --- | --- | --- | --- | --- | --- | --- | --- | --- | --- | --- | --- | --- | --- | --- | --- | --- | --- | --- | --- | --- | --- | --- | --- | --- | --- | --- | --- | --- | --- | --- | --- | --- | --- | --- | --- | --- | --- | --- | --- | --- | --- | --- | --- | --- | --- | --- | --- | --- | --- | --- | --- | --- | --- | --- | --- | --- | --- | --- | --- | --- | --- | --- | --- | --- | --- | --- | --- | --- | --- | --- | --- | --- | --- | --- | --- | --- | --- | --- | --- | --- | --- | --- | --- | --- | --- | --- | --- | --- | --- | --- | --- | --- | --- | --- | --- | --- | --- | --- | --- | --- | --- | --- | --- | --- | --- | --- | --- | --- | --- | --- | --- | --- | --- | --- | --- | --- | --- | --- | --- | --- | --- | --- | --- | --- | --- | --- | --- | --- | --- | --- | --- | --- | --- | --- | --- |

| (B) *Schedule:* Optimal caffeine solution - 5 h per night sleep (morning shift)   | **Sleep** | | | | **Peak Alertness** | | | | **Caffeine** | | | | --- | --- | --- | --- | --- | --- | --- | --- | --- | --- | --- | | **Start** | | **End** | | **Start** | | **End** | | | **Day** | **Time** | **Day** | **Time** | **Day** | **Time** | **Day** | **Time** | **Day** | **Time** | **Dose (mg)** | | 0 | 23:00 | 1 | 07:00 | 2 | 08:00 | 2 | 16:00 | 2 | 07:00 | 100 | | 2 | 01:00 | 2 | 06:00 | 3 | 08:00 | 3 | 16:00 | 2 | 09:00 | 200 | | 3 | 01:00 | 3 | 06:00 | 4 | 08:00 | 4 | 16:00 | 3 | 07:00 | 200 | | 4 | 01:00 | 4 | 06:00 | 5 | 08:00 | 5 | 16:00 | 3 | 09:00 | 200 | | 5 | 01:00 | 5 | 06:00 | 6 | 08:00 | 6 | 16:00 | 4 | 07:00 | 200 | | 6 | 01:00 | 6 | 06:00 |  |  |  |  | 4 | 09:00 | 100 | | 7 | 01:00 | 7 | 09:00 |  |  |  |  | 4 | 11:00 | 100 | |  |  |  |  |  |  |  |  | 5 | 07:00 | 200 | |  |  |  |  |  |  |  |  | 5 | 09:00 | 100 | |  |  |  |  |  |  |  |  | 5 | 11:00 | 100 | |  |  |  |  |  |  |  |  | 6 | 07:00 | 200 | |  |  |  |  |  |  |  |  | 6 | 09:00 | 100 | |  |  |  |  |  |  |  |  | 6 | 12:00 | 100 | |
| --- | --- | --- | --- | --- | --- | --- | --- | --- | --- | --- | --- | --- | --- | --- | --- | --- | --- | --- | --- | --- | --- | --- | --- | --- | --- | --- | --- | --- | --- | --- | --- | --- | --- | --- | --- | --- | --- | --- | --- | --- | --- | --- | --- | --- | --- | --- | --- | --- | --- | --- | --- | --- | --- | --- | --- | --- | --- | --- | --- | --- | --- | --- | --- | --- | --- | --- | --- | --- | --- | --- | --- | --- | --- | --- | --- | --- | --- | --- | --- | --- | --- | --- | --- | --- | --- | --- | --- | --- | --- | --- | --- | --- | --- | --- | --- | --- | --- | --- | --- | --- | --- | --- | --- | --- | --- | --- | --- | --- | --- | --- | --- | --- | --- | --- | --- | --- | --- | --- | --- | --- | --- | --- | --- | --- | --- | --- | --- | --- | --- | --- | --- | --- | --- | --- | --- | --- | --- | --- | --- | --- | --- | --- | --- | --- | --- | --- | --- | --- | --- | --- | --- | --- | --- | --- | --- | --- | --- | --- | --- | --- | --- | --- | --- | --- | --- | --- | --- | --- | --- | --- | --- | --- | --- |

**Figure S3.** Sleep schedule, peak-alertness schedule, and caffeine recommendations for the results depicted in Figure S2. U.S. Army guidelines (a) and optimal caffeine solution automatically generated by *2B-Alert* Web (b). Users export this information as Portable Document Format files by clicking on the left-most of the four buttons on the upper right-hand side of each schedule’s timeline in Figure S2.


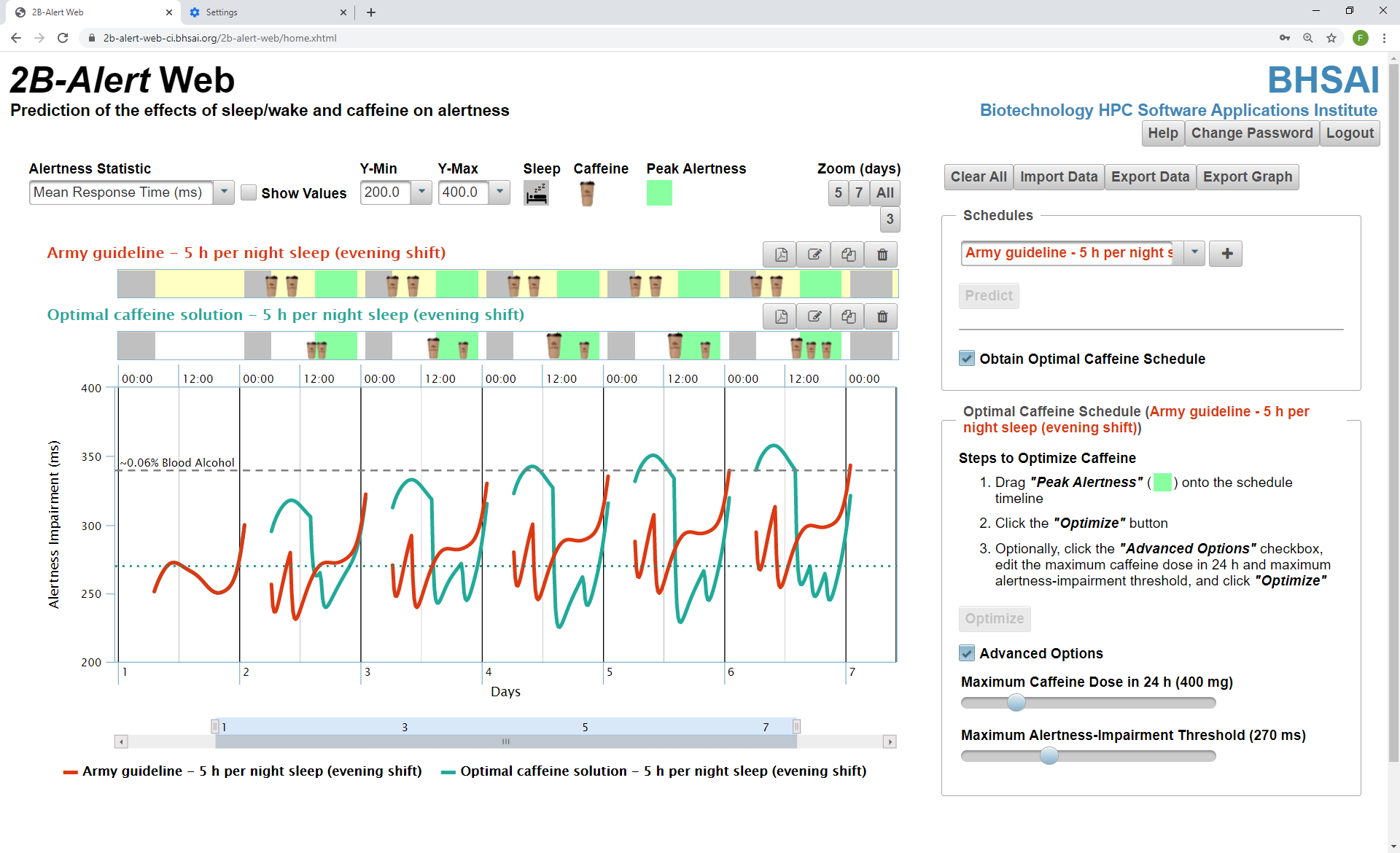


**Figure S4.** *2B-Alert* Web vs. U.S. Army caffeine recommendations for sleep restriction with evening work shift (Condition *3* in Table 1). Comparison of the effects of caffeine countermeasures as recommended by the U.S. Army guidelines (top schedule) vs. those automatically identified by the *2B-Alert* Web tool (bottom schedule) for 5 h per night sleep for 5 nights, with a user-defined peak-alertness period ranging from 15:00 to 23:00 each day. When compared to the U.S. Army guideline, for this peak-alertness period, the optimal caffeine solution provided by the *2B-Alert* Web tool yielded alertness predictions that reduced the mean alertness impairment level by 94% and the peak alertness impairment level by 71% (Table 2).

| (A) Schedule: Army guideline - 5 h per night sleep (evening shift)  | **Sleep** | | | | **Peak Alertness** | | | | **Caffeine** | | | | --- | --- | --- | --- | --- | --- | --- | --- | --- | --- | --- | | **Start** | | **End** | | **Start** | | **End** | | | **Day** | **Time** | **Day** | **Time** | **Day** | **Time** | **Day** | **Time** | **Day** | **Time** | **Dose (mg)** | | 0 | 23:00 | 1 | 07:00 | 2 | 15:00 | 2 | 23:00 | 2 | 06:00 | 200 | | 2 | 01:00 | 2 | 06:00 | 3 | 15:00 | 3 | 23:00 | 2 | 10:00 | 200 | | 3 | 01:00 | 3 | 06:00 | 4 | 15:00 | 4 | 23:00 | 3 | 06:00 | 200 | | 4 | 01:00 | 4 | 06:00 | 5 | 15:00 | 5 | 23:00 | 3 | 10:00 | 200 | | 5 | 01:00 | 5 | 06:00 | 6 | 15:00 | 6 | 23:00 | 4 | 06:00 | 200 | | 6 | 01:00 | 6 | 06:00 |  |  |  |  | 4 | 10:00 | 200 | | 7 | 01:00 | 7 | 09:00 |  |  |  |  | 5 | 06:00 | 200 | |  |  |  |  |  |  |  |  | 5 | 10:00 | 200 | |  |  |  |  |  |  |  |  | 6 | 06:00 | 200 | |  |  |  |  |  |  |  |  | 6 | 10:00 | 200 | |
| --- | --- | --- | --- | --- | --- | --- | --- | --- | --- | --- | --- | --- | --- | --- | --- | --- | --- | --- | --- | --- | --- | --- | --- | --- | --- | --- | --- | --- | --- | --- | --- | --- | --- | --- | --- | --- | --- | --- | --- | --- | --- | --- | --- | --- | --- | --- | --- | --- | --- | --- | --- | --- | --- | --- | --- | --- | --- | --- | --- | --- | --- | --- | --- | --- | --- | --- | --- | --- | --- | --- | --- | --- | --- | --- | --- | --- | --- | --- | --- | --- | --- | --- | --- | --- | --- | --- | --- | --- | --- | --- | --- | --- | --- | --- | --- | --- | --- | --- | --- | --- | --- | --- | --- | --- | --- | --- | --- | --- | --- | --- | --- | --- | --- | --- | --- | --- | --- | --- | --- | --- | --- | --- | --- | --- | --- | --- | --- | --- | --- | --- | --- | --- | --- | --- | --- | --- | --- | --- | --- | --- |

| (B) *Schedule:* Optimal caffeine solution - 5 h per night sleep (evening shift)   | **Sleep** | | | | **Peak Alertness** | | | | **Caffeine** | | | | --- | --- | --- | --- | --- | --- | --- | --- | --- | --- | --- | | **Start** | | **End** | | **Start** | | **End** | | | **Day** | **Time** | **Day** | **Time** | **Day** | **Time** | **Day** | **Time** | **Day** | **Time** | **Dose (mg)** | | 0 | 23:00 | 1 | 07:00 | 2 | 15:00 | 2 | 23:00 | 2 | 14:00 | 100 | | 2 | 01:00 | 2 | 06:00 | 3 | 15:00 | 3 | 23:00 | 2 | 16:00 | 100 | | 3 | 01:00 | 3 | 06:00 | 4 | 15:00 | 4 | 23:00 | 3 | 14:00 | 200 | | 4 | 01:00 | 4 | 06:00 | 5 | 15:00 | 5 | 23:00 | 3 | 20:00 | 100 | | 5 | 01:00 | 5 | 06:00 | 6 | 15:00 | 6 | 23:00 | 4 | 14:00 | 300 | | 6 | 01:00 | 6 | 06:00 |  |  |  |  | 4 | 20:00 | 100 | | 7 | 01:00 | 7 | 09:00 |  |  |  |  | 5 | 14:00 | 300 | |  |  |  |  |  |  |  |  | 5 | 20:00 | 100 | |  |  |  |  |  |  |  |  | 6 | 14:00 | 200 | |  |  |  |  |  |  |  |  | 6 | 17:00 | 100 | |  |  |  |  |  |  |  |  | 6 | 20:00 | 100 | |
| --- | --- | --- | --- | --- | --- | --- | --- | --- | --- | --- | --- | --- | --- | --- | --- | --- | --- | --- | --- | --- | --- | --- | --- | --- | --- | --- | --- | --- | --- | --- | --- | --- | --- | --- | --- | --- | --- | --- | --- | --- | --- | --- | --- | --- | --- | --- | --- | --- | --- | --- | --- | --- | --- | --- | --- | --- | --- | --- | --- | --- | --- | --- | --- | --- | --- | --- | --- | --- | --- | --- | --- | --- | --- | --- | --- | --- | --- | --- | --- | --- | --- | --- | --- | --- | --- | --- | --- | --- | --- | --- | --- | --- | --- | --- | --- | --- | --- | --- | --- | --- | --- | --- | --- | --- | --- | --- | --- | --- | --- | --- | --- | --- | --- | --- | --- | --- | --- | --- | --- | --- | --- | --- | --- | --- | --- | --- | --- | --- | --- | --- | --- | --- | --- | --- | --- | --- | --- | --- | --- | --- | --- | --- | --- | --- | --- | --- | --- | --- | --- | --- | --- |

**Figure S5.** Sleep schedule, peak-alertness schedule, and caffeine recommendations for the results depicted in Figure S4. U.S. Army guidelines (a) and optimal caffeine solution automatically generated by *2B-Alert* Web (b). Users export this information as Portable Document Format files by clicking on the left-most of the four buttons on the upper right-hand side of each schedule’s timeline in Figure S4.


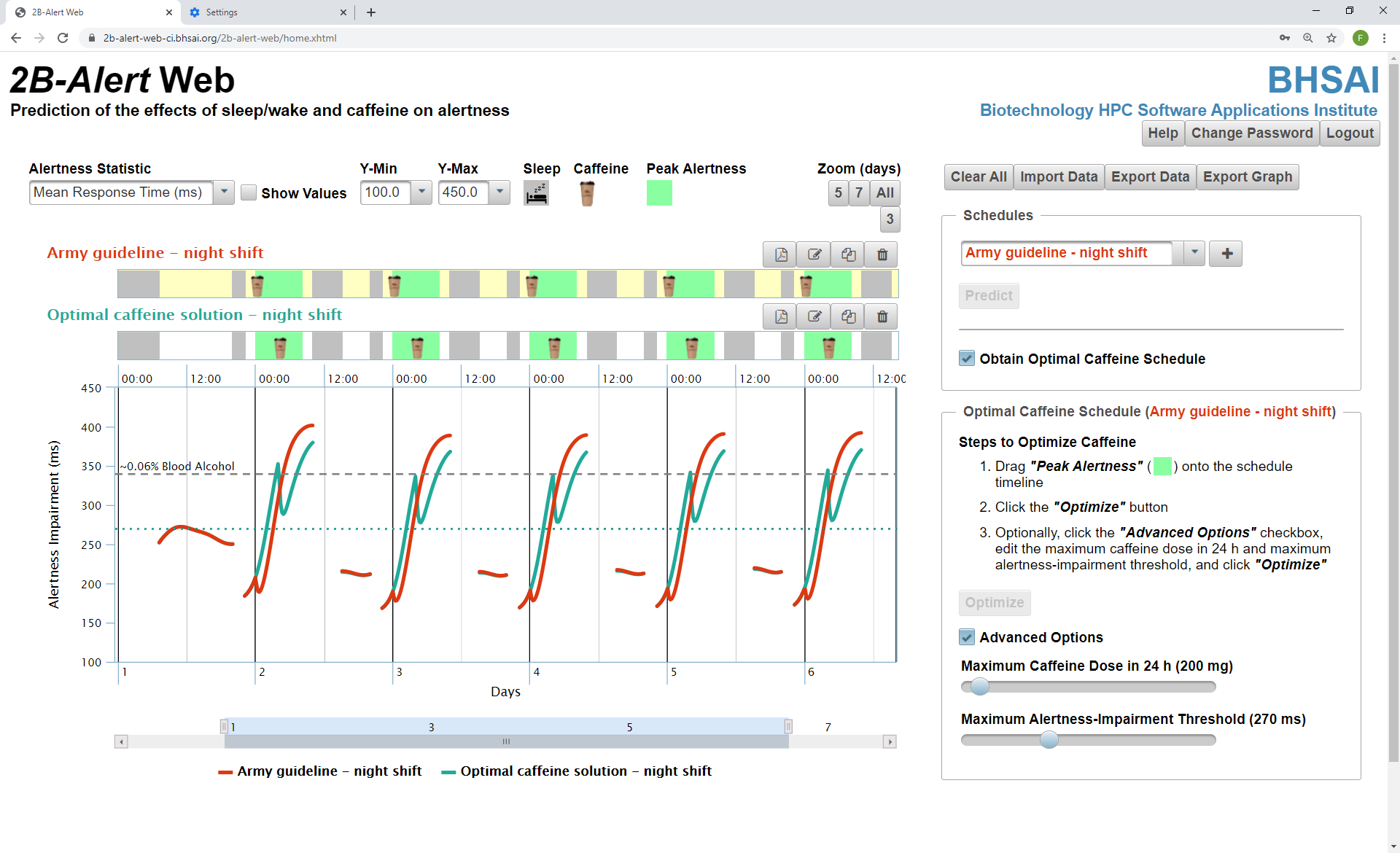


**Figure S6.** *2B-Alert* Web vs. U.S. Army caffeine recommendations for night shift with daytime sleep (Condition *4* in Table 1). Comparison of the effects of caffeine countermeasures as recommended by the U.S. Army guidelines (top schedule) vs. those automatically identified by the *2B-Alert* Web tool (bottom schedule) for night shift, with a user-defined peak-alertness period ranging from 00:00 to 08:00 for each of 5 nights. When compared to the U.S. Army guideline, for this peak-alertness period, the optimal caffeine solution provided by the *2B-Alert* Web tool yielded alertness predictions that reduced the mean alertness impairment level by 36% and the peak alertness impairment level by 31% (Table 2).

| (A) Schedule: Army guideline – night shift  | **Sleep** | | | | **Peak Alertness** | | | | **Caffeine** | | | | --- | --- | --- | --- | --- | --- | --- | --- | --- | --- | --- | | **Start** | | **End** | | **Start** | | **End** | | | **Day** | **Time** | **Day** | **Time** | **Day** | **Time** | **Day** | **Time** | **Day** | **Time** | **Dose (mg)** | | 0 | 23:00 | 1 | 07:00 | 2 | 00:00 | 2 | 08:00 | 2 | 00:00 | 200 | | 1 | 20:00 | 1 | 22:00 | 3 | 00:00 | 3 | 08:00 | 3 | 00:00 | 200 | | 2 | 10:00 | 2 | 15:00 | 4 | 00:00 | 4 | 08:00 | 4 | 00:00 | 200 | | 2 | 20:00 | 2 | 22:00 | 5 | 00:00 | 5 | 08:00 | 5 | 00:00 | 200 | | 3 | 10:00 | 3 | 15:00 | 6 | 00:00 | 6 | 08:00 | 6 | 00:00 | 200 | | 3 | 20:00 | 3 | 22:00 |  |  |  |  |  |  |  | | 4 | 10:00 | 4 | 15:00 |  |  |  |  |  |  |  | | 4 | 20:00 | 4 | 22:00 |  |  |  |  |  |  |  | | 5 | 10:00 | 5 | 15:00 |  |  |  |  |  |  |  | | 5 | 20:00 | 5 | 22:00 |  |  |  |  |  |  |  | | 6 | 10:00 | 6 | 15:00 |  |  |  |  |  |  |  | |
| --- | --- | --- | --- | --- | --- | --- | --- | --- | --- | --- | --- | --- | --- | --- | --- | --- | --- | --- | --- | --- | --- | --- | --- | --- | --- | --- | --- | --- | --- | --- | --- | --- | --- | --- | --- | --- | --- | --- | --- | --- | --- | --- | --- | --- | --- | --- | --- | --- | --- | --- | --- | --- | --- | --- | --- | --- | --- | --- | --- | --- | --- | --- | --- | --- | --- | --- | --- | --- | --- | --- | --- | --- | --- | --- | --- | --- | --- | --- | --- | --- | --- | --- | --- | --- | --- | --- | --- | --- | --- | --- | --- | --- | --- | --- | --- | --- | --- | --- | --- | --- | --- | --- | --- | --- | --- | --- | --- | --- | --- | --- | --- | --- | --- | --- | --- | --- | --- | --- | --- | --- | --- | --- | --- | --- | --- | --- | --- | --- | --- | --- | --- | --- | --- | --- | --- | --- | --- | --- | --- | --- | --- | --- | --- | --- | --- | --- | --- | --- | --- | --- | --- |

| (B) *Schedule:* Optimal caffeine solution - night shift   | **Sleep** | | | | **Peak Alertness** | | | | **Caffeine** | | | | --- | --- | --- | --- | --- | --- | --- | --- | --- | --- | --- | | **Start** | | **End** | | **Start** | | **End** | | | **Day** | **Time** | **Day** | **Time** | **Day** | **Time** | **Day** | **Time** | **Day** | **Time** | **Dose (mg)** | | 0 | 23:00 | 1 | 07:00 | 2 | 00:00 | 2 | 08:00 | 2 | 04:00 | 200 | | 1 | 20:00 | 1 | 22:00 | 3 | 00:00 | 3 | 08:00 | 3 | 04:00 | 200 | | 2 | 10:00 | 2 | 15:00 | 4 | 00:00 | 4 | 08:00 | 4 | 04:00 | 200 | | 2 | 20:00 | 2 | 22:00 | 5 | 00:00 | 5 | 08:00 | 5 | 04:00 | 200 | | 3 | 10:00 | 3 | 15:00 | 6 | 00:00 | 6 | 08:00 | 6 | 04:00 | 200 | | 3 | 20:00 | 3 | 22:00 |  |  |  |  |  |  |  | | 4 | 10:00 | 4 | 15:00 |  |  |  |  |  |  |  | | 4 | 20:00 | 4 | 22:00 |  |  |  |  |  |  |  | | 5 | 10:00 | 5 | 15:00 |  |  |  |  |  |  |  | | 5 | 20:00 | 5 | 22:00 |  |  |  |  |  |  |  | | 6 | 10:00 | 6 | 15:00 |  |  |  |  |  |  |  | |
| --- | --- | --- | --- | --- | --- | --- | --- | --- | --- | --- | --- | --- | --- | --- | --- | --- | --- | --- | --- | --- | --- | --- | --- | --- | --- | --- | --- | --- | --- | --- | --- | --- | --- | --- | --- | --- | --- | --- | --- | --- | --- | --- | --- | --- | --- | --- | --- | --- | --- | --- | --- | --- | --- | --- | --- | --- | --- | --- | --- | --- | --- | --- | --- | --- | --- | --- | --- | --- | --- | --- | --- | --- | --- | --- | --- | --- | --- | --- | --- | --- | --- | --- | --- | --- | --- | --- | --- | --- | --- | --- | --- | --- | --- | --- | --- | --- | --- | --- | --- | --- | --- | --- | --- | --- | --- | --- | --- | --- | --- | --- | --- | --- | --- | --- | --- | --- | --- | --- | --- | --- | --- | --- | --- | --- | --- | --- | --- | --- | --- | --- | --- | --- | --- | --- | --- | --- | --- | --- | --- | --- | --- | --- | --- | --- | --- | --- | --- | --- | --- | --- | --- |

**Figure S7.** Sleep schedule, peak-alertness schedule, and caffeine recommendations for the results depicted in Figure S6. U.S. Army guidelines (a) and optimal caffeine solution automatically generated by *2B-Alert* Web (b). Users export this information as Portable Document Format files by clicking on the left-most of the four buttons on the upper right-hand side of each schedule’s timeline in Figure S6.
